# Genome-wide association study-based prediction of atrial fibrillation using artificial intelligence

**DOI:** 10.1136/openhrt-2021-001898

**Published:** 2022-01-27

**Authors:** Oh-Seok Kwon, Myunghee Hong, Tae-Hoon Kim, Inseok Hwang, Jaemin Shim, Eue-Keun Choi, Hong Euy Lim, Hee Tae Yu, Jae-Sun Uhm, Boyoung Joung, Seil Oh, Moon-Hyoung Lee, Young-Hoon Kim, Hui-Nam Pak

**Affiliations:** 1 Cardiology, Yonsei University Health System, Seodaemun-gu, Seoul, Korea (the Republic of); 2 Cardiovascular Center, Korea University Medical Center, Seoul, Korea (the Republic of); 3 Cardiology, Seoul National University, Seoul, Korea (the Republic of); 4 Cardiology, Hallym University Sacred Heart Hospital, Anyang, Gyeonggi-do, Korea (the Republic of)

**Keywords:** genome-wide association study, atrial fibrillation, genetics

## Abstract

**Objective:**

We previously reported early-onset atrial fibrillation (AF) associated genetic loci among a Korean population. We explored whether the AF-associated single-nucleotide polymorphisms (SNPs) selected from the Genome-Wide Association Study (GWAS) of an external large cohort has a prediction power for AF in Korean population through a convolutional neural network (CNN).

**Methods:**

This study included 6358 subjects (872 cases, 5486 controls) from the Korean population GWAS data. We extracted the lists of SNPs at each p value threshold of the association statistics from three different previously reported ethnical-specific GWASs. The Korean GWAS data were divided into training (64%), validation (16%) and test (20%) sets, and a stratified K-fold cross-validation was performed and repeated five times after data shuffling.

**Results:**

The CNN-GWAS predictive power for AF had an area under the curve (AUC) of 0.78±0.01 based on the Japanese GWAS, AUC of 0.79±0.01 based on the European GWAS, and AUC of 0.82±0.01 based on the multiethnic GWAS, respectively. Gradient-weighted class activation mapping assigned high saliency scores for AF associated SNPs, and the *PITX2* obtained the highest saliency score. The CNN-GWAS did not show AF prediction power by SNPs with non-significant p value subset (AUC 0.56±0.01) despite larger numbers of SNPs. The CNN-GWAS had no prediction power for odd–even registration numbers (AUC 0.51±0.01).

**Conclusions:**

AF can be predicted by genetic information alone with moderate accuracy. The CNN-GWAS can be a robust and useful tool for detecting polygenic diseases by capturing the cumulative effects and genetic interactions of moderately associated but statistically significant SNPs.

**Trial registration number:**

NCT02138695.

**Key questions**

**What is already known about this subject?**

- Atrial fibrillation (AF) is known to be a heritable disease, and multiple genetic loci associated with AF have been reported by genome-wide association study (GWAS) studies.

**What does this study add?**

- The collaborative method incorporating a convolutional neural network (CNN) and GWAS could classify the AF vs non-AF with genetic information alone.
- CNN-GWAS with explainable artificial intelligence technique provides a new perspective for GWAS by identifying the positive and negative interactions of each single-nucleotide polymorphism (SNP).

**How might this impact on clinical practice?**

- CNN-GWAS can be a robust method to predict AF patients by highlighting the cumulative effects and genetic interactions of moderately associated, but statistically significant SNPs. Further studies of comparison and validation with other predictive models are needed to standardised testing.

## Introduction

Atrial fibrillation (AF) is a major cardiovascular disease with a prevalence of 1.6% in the total population and is the cause of 20%–25% of ischaemic strokes and about 30% of heart failure.^1^ AF is a chronic degenerative disease that progresses from a paroxysmal to persistent type, long-standing persistent and permanent AF.^2^ As more than 50% of AF occurs asymptomatically, early-stage low burden paroxysmal AF is difficult to diagnose by a single examination with an ECG.^3^ Moreover, after progressing to persistent AF, rhythm control becomes more difficult than in the paroxysmal AF stage, and the recurrence rate is significantly increased.^2^ Therefore, it is practical to prevent AF progression or its related complications by an early diagnosis or predicting the occurrence of AF. AF is known to be a heritable disease, and the risk of AF increases by more than 40% if a parent or sibling has AF.^4^ As the Genome-Wide Association Study (GWAS) has become popular in research, multiple genetic loci related to AF have been reported.^5^ However, it is difficult to find a rare variant gene, and the contribution of genes with intermediate specificity can be neglected because of the high specificity of the GWAS.^6^ In addition, the genome-wide analysis computes a large amount of genetic information using complex statistical techniques, and therefore, a long and complicated analytic process by a population genetics expert is essential. Because of these technical limitations, the research on the convergence of genetic and clinical information has largely been conducted by multicentre consortiums and serves as a hurdle to the consistent use of the GWAS data in clinical medicine.^7^ As artificial intelligence (AI) research has become more common and popular, the convolutional neural network (CNN) analysis of large-scale genetic data is expected to be faster, more efficient and accurate, but difficulties in interpreting the results still exist.^8^ In this study, we applied CNN and gradient-weighted class activation mapping (Grad-CAM)^9^ to the GWAS analysis to evaluate the effectiveness and accuracy of the method in the prediction power of AF using genomic data. After categorising AF associated single-nucleotide polymorphisms (SNPs) based on the p values taken from previously reported GWAS summary statistics (Japanese,^10^ European^11^ and multiethnic^12^ studies) independent of the Korean GWAS dataset, we conducted a CNN-GWAS after SNPs encoding with a minor allele. We evaluated the prediction power of the CNN-GWAS and verified it in four different ways including Grad-CAM. The purpose of this study was to investigate the potential of AI as a tool for the clinical use in the early diagnosis and risk prediction by using genetic information. We also compared AI selected genomes and the early-onset AF associated genetic loci published in our group based on the same GWAS cohort database.^5^

## Methods

### Study design and subjects

**Figure 1:**
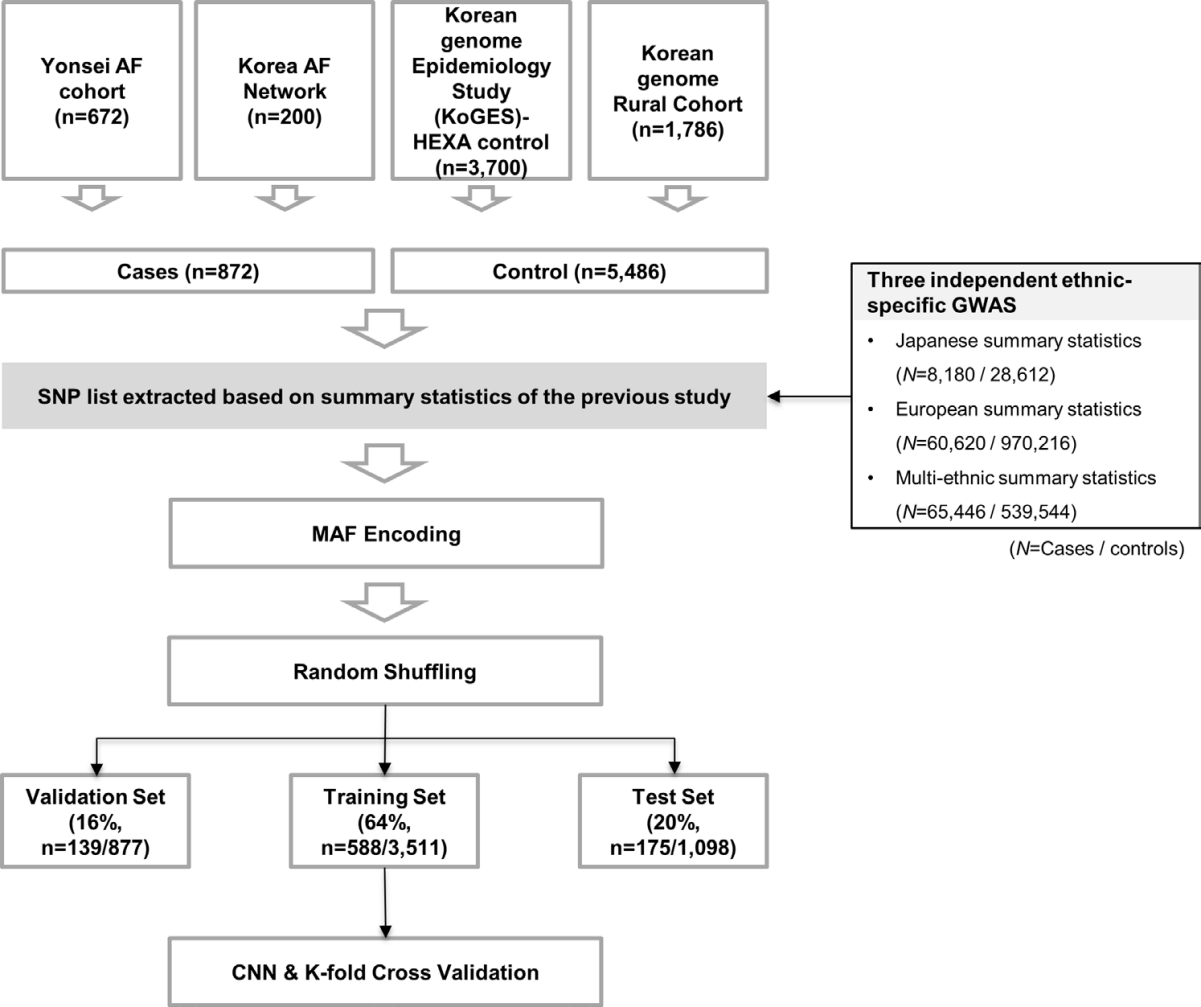
Study flow chart showing the process of the CNN-GWAS, including the AF data set. AF, atrial fibrillation; CNN, convolutional neural network; GWAS, Genome-Wide Association Study; HEXA, health examinee; KoGES, korea genome epidemiology study; MAF, minor allele frequency; SNP, single-nucleotide polymorphism.

This study protocol adhered to the principles of the Declaration of Helsinki. We included 6358 subjects from four independent cohorts and their GWAS data (figure 1). The case group consisted of 872 patients with early-onset AF (<60 years old), who underwent AF catheter ablation and had GWAS data available, recruited from the Yonsei AF ablation cohort (n=672) and Korean AF Network (n=200, figure 1).A detailed description is available in the online supplemental material.

10.1136/openhrt-2021-001898.supp1 Supplementary data

### Genotyping

Samples in the genetic dataset were used in our previously published early-onset AF GWAS.^5^ All subjects were extracted the genomic DNA from peripheral blood monocytes by standard procedures and genotyped by the Affymetrix Genome-Wide Human SNP Array V.6.0 chip (Affymetrix, Santa Clara, California, USA). A detailed description is available in online supplemental material.

### Preprocessing with sampling based on the previously published GWAS

**Figure 2:**
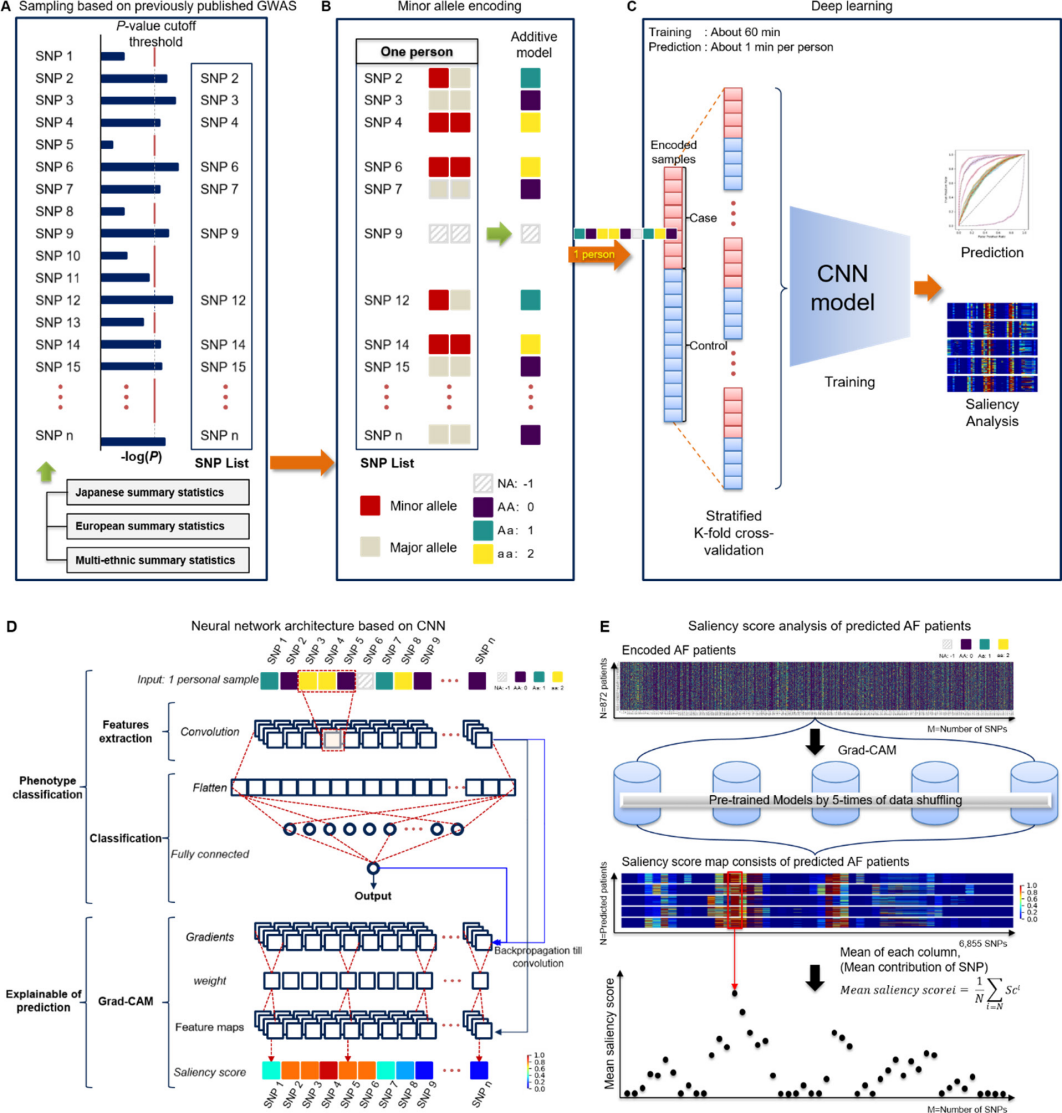
Overview of the CNN-GWAS based framework. (A) A previously reported GWAS-based sampling extracted a set of SNPs according to the p value cut-off. (B) Digitisation encoding for heterozygous or homozygous by minor alleles. (C) Process of the CNN-based neural network model prediction and analysis. (D) Neural network architecture based on the CNN. (F) Saliency score analysis of the predicted AF patients. The same AF patients (n=872) were used for the quantitative evaluation of the five-time pretrained models using different samples. AF, atrial fibrillation; CNN, convolutional neural network; Grad-CAM, gradient-weighted class activation mapping; GWAS, Genome-Wide Association Study; SNP, single-nucleotide polymorphism.

Too many inputs can cause overfitting,^13^ so we needed the feature selection to remove unnecessary SNPs. Therefore, we preselected from the previously published external GWAS (Japanese,^10^ European^11^ and multiethnic^12^ population cohorts) to ensure reliability and independence (online supplemental table 1). Our total number of SNPs was 531 766, and the numbers of common SNPs mapped to our SNPs were 471 462 for Japanese, 530 847 for European and 528 039 for multiethnic cohorts, respectively. The set of variants reaching each threshold, ranging from a genome-wide significance level of a p<5.0×10^−8^ to p<0.001, was considered as a feature selection prior to model training (figure 2A).

### Minor allele encoding

For machine learning (ML), we coded each SNP with homozygous aa 2, heterozygous Aa 1, and AA 0 for the minor allele as an additive model. The missing genotypes were represented as −1.^14^ That is, the genotype of each locus was set as the input $X_{mn}\in \left\{-1,0,1,2\right\}$ for neurons, where m is the index in the sample (1≤m≤M,M=thenumberofsamples) and n is the n-th SNP of the m-th sample (figure 2B).

### Network model design

We developed a CNN-based model of a hierarchical network so that it can be classified by a locus associated with AF (figure 2A–C). The application of CNN was possible because it can be controlled with image-like properties in that SNPs are arranged on the same physical base pair. Our network model consisted of two hidden layers. The dimension of the input is the [the number of SNP ×1]. The first layer consisted of a convolutional layer for a feature extract at each SNP level, while the second layer combined into a fully connected layer to perform classification by the associated pattern with the phenotype of AF. The full network is shown in figure 2D. A detailed description is available in online supplemental material.

### CNN-GWAS model training

Early stop and drop-out methods were used to avoid overfitting. Further description is available in online supplemental material.

### CNN-GWAS verification

To verify our model, four validation processes were conducted. First, we repeated the training, validation and test processes five times to demonstrate the reproducibility of the AF prediction and each sample was randomly constructed. Second, to examine whether SNPs of statistically non-significant p values by a logistic regression did not really affect the AF prediction, an SNP list was constructed and verified based on a p≥0.99. Third, in order to identify that there was no predictive power for a phenotype without heritability (here are odd–even registration numbers) other than AF, the validity was verified by replacing the AF label with an odd–even registration number. Fourth, the saliency score of each SNP for AF prediction was analysed in all AF patients (n=872) using a model of best-performance among the model (figure 2E). A Grad-CAM was applied to calculate the contribution score of each SNP for the AF prediction of the individual. Fifth, to identify whether the issue by class imbalance affected the AF prediction, we conducted a propensity-score matching study.

Further description is available in online supplemental material.

### Derivation of polygenic risk score

To verify the robustness of CNN-GWAS in determining the AF risk, we evaluated the polygenic risk score (PRS), which is a conventional quantitative metric for the genetic risk.^15^ The PRS was calculated using PLINK software from the same summary statistics as CNN-GWAS and additional criteria for PRS were as follows: removal of SNPs with *r^2^* >0.1 for linkage disequilibrium-based clumping within 250 kb range of the index SNP.

### Model performance evaluation and statistical analyses

The data set consisted of mutually exclusive samples with training (64%), validation (16%) and test (20%) sets, each set was selected at random and directly proportional to the number of cases/controls in the population. The final output probability 0 to 1 of a model designed as a binary classifier was evaluated by the phenotype label Y = (control: 0 or AF patient: 1). The evaluation metrics used the area under the curve (AUC), sensitivity, specificity, positive predictive value, negative predictive value, Gini coefficient,^16^ log-loss and mean square error (MSE). The statistical analyses were performed using R (V.3.6.2) and PLINK software (V.1.9). We also implemented and evaluated the conventional ML methods to compare with the CNN models. We used Bayesian neural network,^17^ Lasso, Ridge and logistic regression to consider the classification problem, and this was developed with a Tensorflow backend. For the Bayesian neural network, the Monte Carlo drop-out rate of 0.5 was applied.

### Patient and public involvement

Patients and/or the public were not involved in the design, or conduct, or reporting, or dissemination plans of this research.

## Results

### Baseline characteristics

Table 1 summarises the characteristics of the case and control groups in four different cohorts. In 872 AF patients who underwent AF catheter ablation, 581 patients (66.6%) had paroxysmal AF. The mean age was significantly lower (50.4±7.9 years old vs 55.6±8.6 years old, p<0.001) and the proportion of males was significantly higher (80.5% vs 45.5%, p<0.001) in the case group than in the control group.

**Table 1:** Characteristics of the GWAS dataset subjects

Baseline characteristics	Combined		Case		Control	
	Case (N=872)	Control (N=5486)	Yonsei AF cohort (N=672)	Korean AF network (N=200)	KoGES-HEXA cohort (N=3700)	Korean genomic rural cohort (N=1786)
Age, year	50.4±7.9	55.6±8.6*	50.5±7.8	50.1±8.2	53.1±8.3	60.7±6.6
Male sex, %	702 (80.5)	2495 (45.5)*	546 (81.3)	156 (78.0)	1649 (44.6)	846 (47.4)
PAF, %	581 (66.6)	–	482 (71.7)	99 (49.5)	–	–
Body mass index, kg/m^2^	25.0±3.0	24.2±3.1*	25.1±3.0	24.8±2.8	24.0±2.9	24.7±3.3
Hypertension, %	303 (34.7)	1278 (23.3)*	237 (35.3)	66 (33.0)	691 (18.7)	587 (32.9)
Diabetes, %	66 (7.6)	899 (16.4)*	51 (7.6)	15 (7.5)	249 (6.7)	650 (36.4)
Coronary artery disease, %	77 (8.8)	149 (2.7)*	56 (8.3)	21 (10.5)	106 (2.9)	43 (2.4)
Stroke, %	54 (6.2)	115 (2.1)*	47 (7.0)	7 (3.5)	56 (1.5)	59 (3.3)

Data are shown as the mean±SD or n (%).

*P<0.05.

AF, atrial fibrillation; GWAS, Genome-Wide Association Study; HEXA, health examinee; KoGES, korea genome epidemiology study; PAF, paroxysmal atrial fibrillation.

### CNN-GWAS prediction model and performance

**Figure 3:**
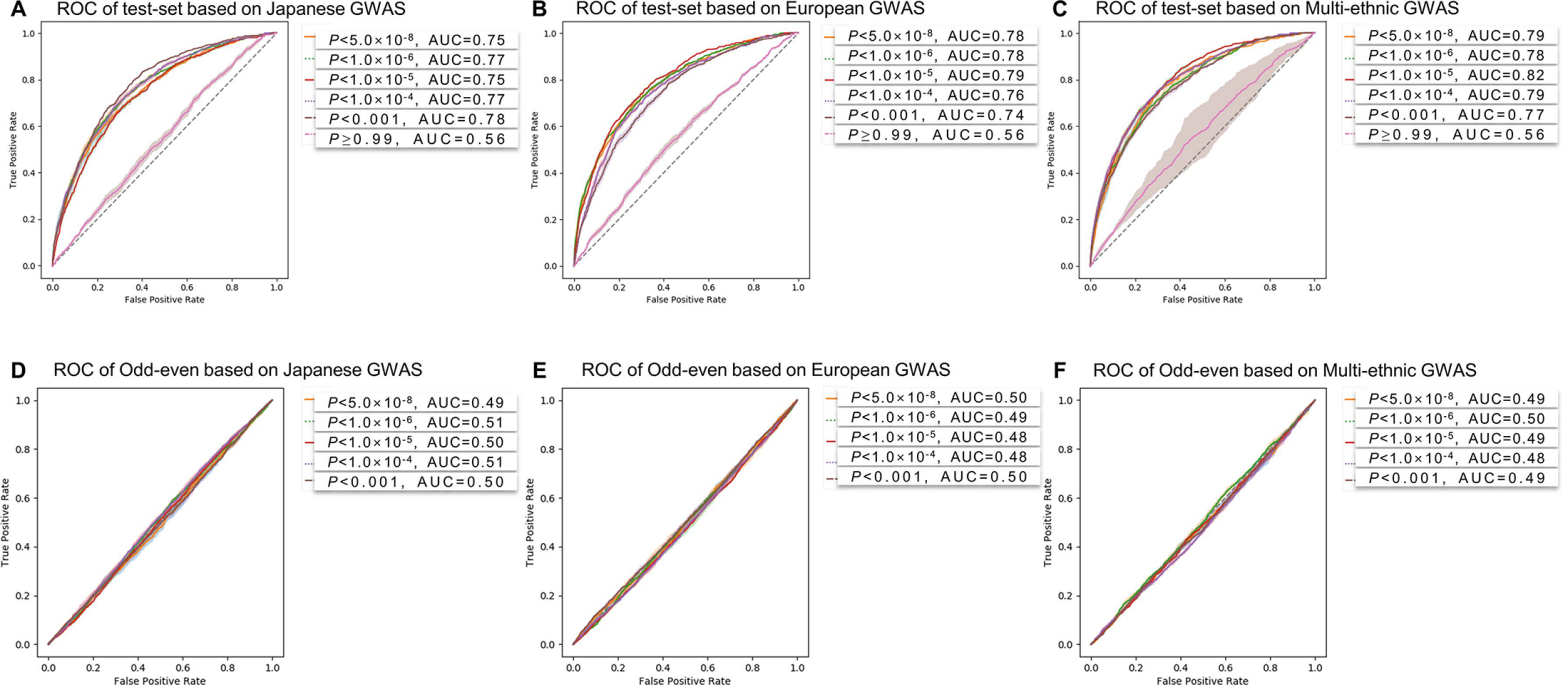
Performance evaluation results. (A–C) The results of the AF prediction ROC curves of the Korean GWAS at each p value cut-off based on the selected SNP set by three different GWAS cohorts’ summary statistics (Japanese, European and multiethnic GWAS). P value cutoffs from a p<0.001 to p<5.0×10^−8^ were used for the performance evaluation, and a p≥0.99 was used for the verification of the non-significant SNP list. (D–F) The prediction results for the odd-even registration numbers with the SNP list for the AF prediction (p value cut-off threshold p<0.001 to p<5.0×10^−8^). All results were repeated five times, and the shaded area shows the 95% CI. AF, atrial fibrillation; AUC, area under the curve; GWAS, Genome-Wide Association Study; ROC, receiver operating characteristic; SNP, single-nucleotide polymorphism.

**Figure 4:**
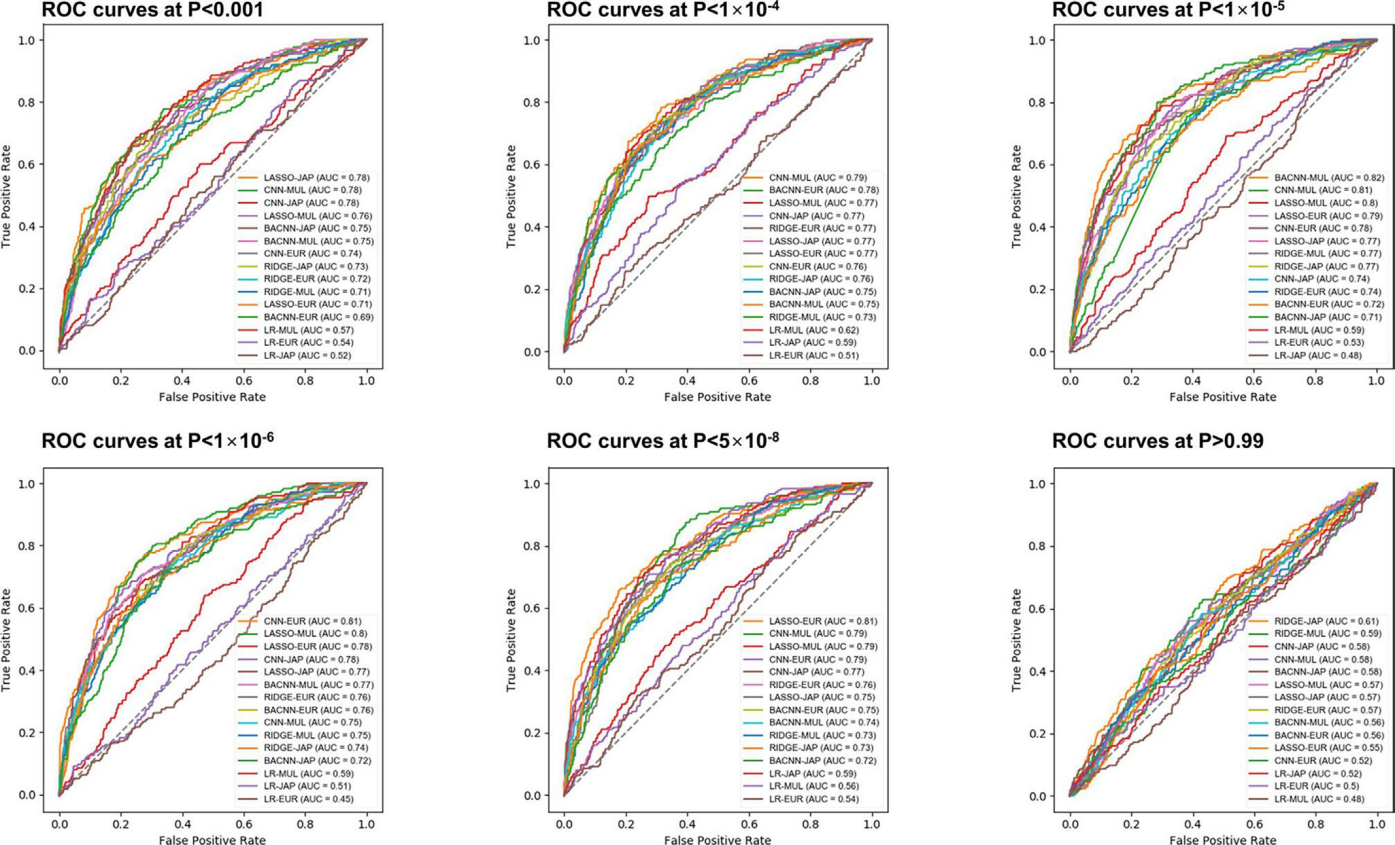
Performance comparison between CNN-GWAS and other machine learning methods. AUC; area under the curve, BACNN; Bayesian approximation convolutional neural network; CNN; convolutional neural network; Eur; European; GWAS, Genome-Wide Association Study; JAP; Japanese; LASSO; least absolute shrinkage and selection operator; LR; logistic regression; MUL; multiethnic; ROC; receiver operating characteristic.

The model training time was about 60 min to learn and the time required to predict the AF risk of an individual was approximately 1 min (figure 2C). The training, validation and test set consisted of randomly selected samples, and all tests were repeated five times. Table 2 shows the mean performance results for the AF predictions in the test sets. The AUC values were 0.78±0.01 for Japanese at p<0.001, 0.79±0.01 for European, and 0.82±0.01 for multiethnic cohorts at a p<1.0×10^−5^, respectively (figure 3A–C). The highest AUC values in each independent cohort are summarised in online supplemental table 3. The receiver operating characteristic (ROC) curve of the validation set is shown in online supplemental figure 2. In addition, there were no significant differences in comparison of the Bayesian neural network, Lasso and Ridge, but logistic regression showed remarkably less predictive power (figure 4).

**Table 2:** Mean predictive performance of the test set

Population type	P value	SNPs	AUC	Sens	Spec	PPV	NPV	Gini	Log-loss	MSE
Japanese	**<0.001**	2211	**0.78±0.01**	**0.73±0.05**	**0.72±0.04**	**0.29±0.02**	**0.94±0.01**	**0.57±0.02**	**1.06±0.80**	**0.18±0.14**
	<1.0×10^−4^	587	0.77±0.01	0.71±0.01	0.71±0.02	0.28±0.01	0.94±0.00	0.54±0.02	0.73±0.07	0.14±0.01
	<1.0×10^−5^	262	0.75±0.01	0.70±0.01	0.69±0.03	0.26±0.02	0.94±0.00	0.50±0.02	0.73±0.11	0.18±0.02
	<1.0×10^−6^	153	0.77±0.01	0.72±0.03	0.69±0.04	0.27±0.02	0.94±0.00	0.54±0.01	0.64±0.03	0.18±0.01
	<5.0×10^−8^	91	0.75±0.01	0.68±0.01	0.70±0.03	0.27±0.01	0.93±0.00	0.50±0.02	0.61±0.01	0.20±0.00
	≥0.990	4221	0.56±0.03	0.54±0.07	0.56±0.04	0.16±0.01	0.88±0.01	0.12±0.05	0.98±0.09	0.16±0.07
European	<0.001	5401	0.74±0.02	0.72±0.05	0.65±0.04	0.25±0.02	0.94±0.01	0.48±0.05	5.46±0.66	0.42±0.27
	<1.0×10^−4^	2755	0.76±0.02	0.72±0.04	0.69±0.04	0.27±0.02	0.94±0.01	0.52±0.04	3.60±1.62	0.61±0.28
	**<1.0×10^−5^**	1704	**0.79±0.01**	**0.74±0.03**	**0.72±0.04**	**0.30±0.02**	**0.95±0.01**	**0.59±0.03**	**0.63±0.05**	**0.12±0.02**
	<1.0×10^−6^	1192	0.78±0.01	0.72±0.04	0.71±0.04	0.29±0.02	0.94±0.01	0.57±0.03	0.72±0.12	0.13±0.04
	<5.0×10^−8^	814	0.78±0.01	0.71±0.03	0.72±0.03	0.29±0.02	0.94±0.00	0.56±0.02	0.68±0.04	0.13±0.03
	≥0.990	4699	0.56±0.05	0.57±0.03	0.55±0.08	0.17±0.02	0.89±0.01	0.12±0.10	2.16±0.73	0.12±0.09
Multiethnic	<0.001	4732	0.77±0.01	0.72±0.05	0.70±0.04	0.27±0.02	0.94±0.01	0.54±0.02	2.39±1.61	0.29±0.34
	<1.0×10^−4^	2372	0.79±0.01	0.72±0.03	0.73±0.04	0.30±0.02	0.94±0.00	0.58±0.03	1.19±0.72	0.13±0.10
	**<1.0×10^−5^**	1540	**0.82±0.01**	**0.74±0.04**	**0.76±0.03**	**0.33±0.01**	**0.95±0.01**	**0.63±0.02**	**0.61±0.07**	**0.12±0.04**
	<1.0×10^−6^	1037	0.78±0.02	0.72±0.03	0.70±0.04	0.28±0.02	0.94±0.01	0.56±0.04	0.81±0.16	0.13±0.01
	<5.0×10^−8^	723	0.79±0.01	0.74±0.03	0.71±0.03	0.29±0.02	0.95±0.00	0.58±0.03	0.75±0.02	0.15±0.03
	≥0.990	4965	0.56±0.01	0.56±0.02	0.56±0.04	0.17±0.01	0.89±0.01	0.12±0.03	0.98±0.09	0.11±0.07

Data are shown as the mean±SD.

The best model of each ethnicity is shown in bold.

AUC, area under the curve; MSE, mean square error; NPV, negative predictive value; PPV, positive predictive value; Sens, sensitivity; SNPs, single-nucleotide polymorphisms; Spec, specificity.

### Model validation with non-significant genomes

To confirm the validity of the p value cut-off for SNP selection, we conversely evaluated the trained model by selecting SNPs with no statistical association. The SNPs without statistically significant association with AF (cut-off p≥0.99) were selected in each ethnic-specific GWAS (4221 SNPs in Japanese, 4699 SNPs in European and 4965 SNPs in multiethnic GWAS). Results using these statistically non-significant associated SNPs showed a poor predictive power for AF (AUC 0.56, figure 3A–C). The AF prediction performance estimated by the sensitivity, or specificity, or Gini coefficient were consistently very low (table 2).

### Model validation by odd–even registration numbers

To evaluate the robustness of the CNN-GWAS model, we tested whether the AF associated SNPs could predict odd or even registration numbers of the included population. The numbers of cases and controls separated by odd–even registration numbers were 3189 and 3169, respectively. The age (54.8±8.7 vs 54.9±8.8 years old, p=0.799) and the proportion of males (50.0% vs 50.6%, p=0.633) did not significantly differ between the two groups. The ROC curve for odd–even registration numbers did not show any predicted values regardless of the p value cut-off, and the variation was also small (figure 3D–F and online supplemental table 4).

### Explanation for an AF prediction using the Grad-CAM

**Figure 5:**
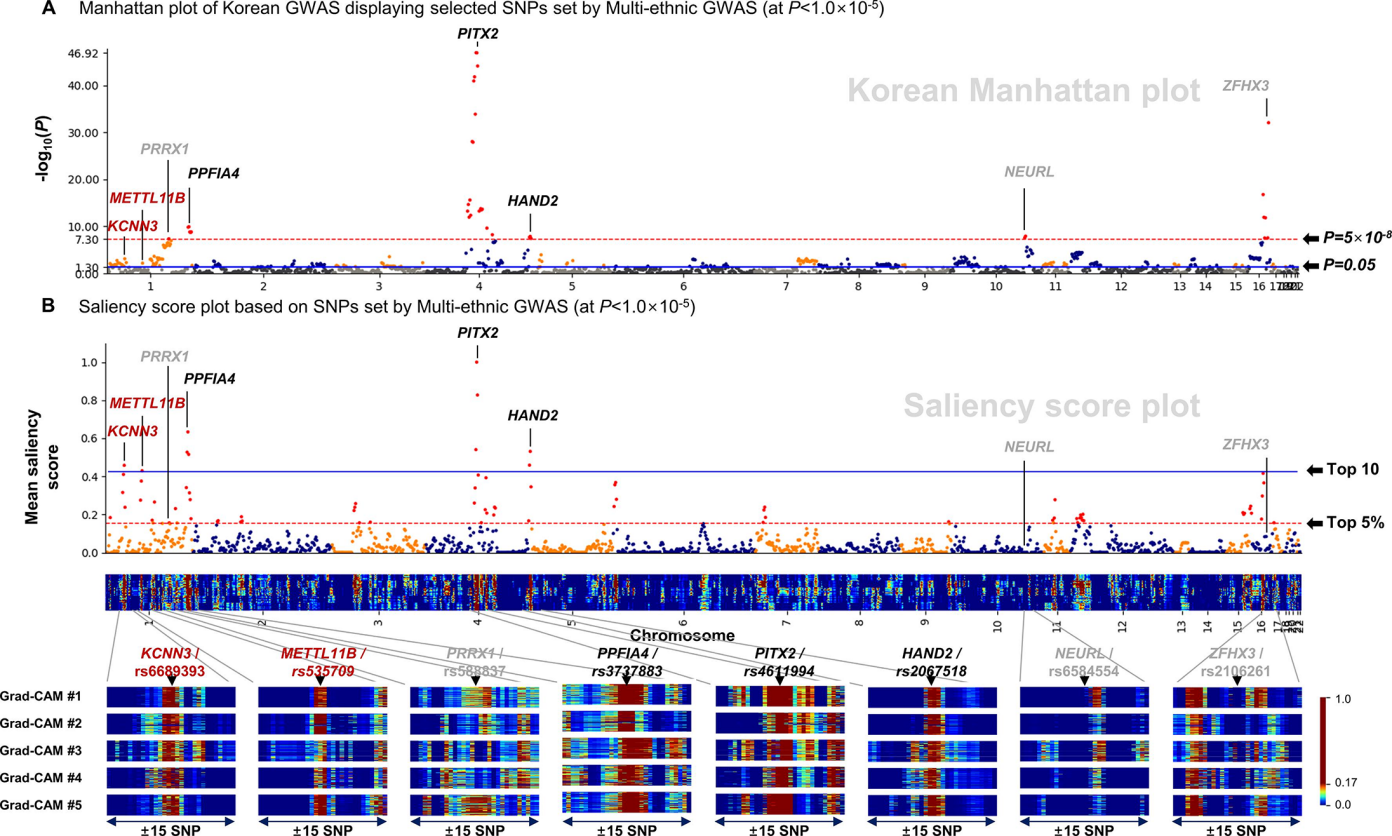
Explanation of the predictive power of the CNN-GWAS for AF. (A) The Manhattan plot of the Korean population GWAS represented by the SNP set selected at a p<1.0×10^−5^ in the multiethnic GWAS. (B) The contribution of each SNP for the AF prediction is represented by a plot (top), which is the mean saliency score for each column of the two-dimensional (2D) saliency score map. The saliency scores of each AF patient are displayed stacked in the 2D saliency score map (below). Those in the grey font were reported to be AF associated SNPs but were not included in the top 10 highest salience scored SNPs. The blue horizontal line stands for the top 10 saliency score levels, and the red dotted horizontal line stands for the top 5% high saliency score levels. AF, atrial fibrillation; CNN, convolutional neural network; Grad-CAM, gradient-weighted class activation mapping; GWAS, Genome-Wide Association Study; SNP, single-nucleotide polymorphism.

We listed the top 10 SNPs with the highest saliency scores analysed by the Grad-CAM analyses in table 3. The *PITX2*, which has been reported as the top first AF associated gene, exhibited a reproducibly with the highest saliency scores in all three independent cohorts of different ethnicities. The other proven AF associated SNPs, such as *KCNN3, METTL11B, PPFIA4, HAND2 and TUBA8*, were also included in the top 10 highest saliency scores. The Pearson correlation coefficient was 0.472 when comparing the Manhattan plot and Saliency score plot, and those displayed the selected SNPs set by the multiethnic GWAS at a p<1.0×10^−5^ (figure 5). In the Manhattan plot (figure 5A), 15 of 36 significant AF-associated SNPs with a genome-wide significance (p<5.0×10^−8^) were ranked in the top 5% of the saliency score plot. Conversely, 75 out of 78 SNPs ranked in the top 5% of the saliency score plot (figure 5B) were previously proven AF associated genetic loci in the multiethnic cohort, of which 54 SNPs were replicated in the Korean GWAS at a p<0.05 level (figure 5A).

**Table 3:** Genetic loci with the top 10 mean saliency scores at the high performance p value threshold of each ethnicity

Chr.	SNP	Position	Closest gene	Minor/major allele	MAF (%)	OR	95% CI	P value	Mean saliency score
Based on Japanese GWAS									
4	rs4611994	111 711 041	PITX2*	T/C	47.64	0.42	0.37 to 0.47	1.29×10^−47^	1.000
4	rs17042171	111 708 287	PITX2*	C/A	47.66	0.42	0.37 to 0.47	1.20×10^−47^	0.711
4	rs6843082	111 718 067	PITX2*	A/G	27.21	0.32	0.27 to 0.37	8.06×10^−45^	0.708
1	rs3737883	203 034 906	PPFIA4*	G/A	31.75	0.67	0.59 to 0.75	1.22×10^−10^	0.498
4	rs6852021	111 744 112	PITX2*	A/G	49.58	0.79	0.71 to 0.88	1.37×10^−5^	0.474
1	rs11579055	203 031 315	PPFIA4*	T/G	32.26	0.67	0.59 to 0.76	1.54×10^−10^	0.434
4	rs723364	111 724 471	PITX2*	G/C	20.32	0.82	0.71 to 0.95	0.006	0.421
4	rs17042144	111 689 666	PITX2*	C/T	42.31	2.01	1.80 to 2.24	1.40×10^−34^	0.403
1	rs6694477	203 035 365	PPFIA4*	G/A	21.05	0.64	0.55 to 0.74	1.53×10^−9^	0.386
20	rs11696871	62 387 417	*ZBTB46*	A/G	48.64	0.94	0.84 to 1.04	0.242	0.385
Based on European GWAS									
4	rs4611994	111 711 041	PITX2*	T/C	47.64	0.42	0.37 to 0.47	1.29×10^−47^	1.000
4	rs17042171	111 708 287	PITX2*	C/A	47.66	0.42	0.37 to 0.47	1.20×10^−47^	0.740
4	rs6843082	111 718 067	PITX2*	A/G	27.21	0.32	0.27 to 0.37	8.06×10^−45^	0.716
1	rs11579055	203 031 315	PPFIA4*	T/G	32.26	0.67	0.59 to 0.76	1.54×10^−10^	0.488
1	rs3737883	203 034 906	PPFIA4*	G/A	31.75	0.67	0.59 to 0.75	1.22×10^−10^	0.486
22	rs464385	18 571 008	TUBA8*	A/G	43.12	1.01	0.91 to 1.13	0.824	0.485
4	rs17042144	111 689 666	PITX2*	C/T	42.31	2.01	1.80 to 2.24	1.40×10^−34^	0.457
22	rs361594	18 577 338	TUBA8*	T/C	48.19	0.91	0.82 to 1.02	0.105	0.440
1	rs6694477	203 035 365	PPFIA4*	G/A	21.05	0.64	0.55 to 0.74	1.53×10^−9^	0.401
4	rs3866838	111 753 815	PITX2*	T/C	17.75	0.85	0.73 to 0.98	0.025	0.399
Based on multi-ethnic GWAS									
4	rs4611994	111 711 041	PITX2*	T/C	47.64	0.42	0.37 to 0.47	1.29×10^−47^	1.000
4	rs6843082	111 718 067	PITX2*	A/G	27.21	0.32	0.27 to 0.37	8.06×10^−45^	0.827
1	rs3737883	203 034 906	PPFIA4*	G/A	31.75	0.67	0.59 to 0.75	1.22×10^−10^	0.634
4	rs17042171	111 708 287	PITX2*	C/A	47.66	0.42	0.37 to 0.47	1.20×10^−47^	0.540
4	rs2067518	174 612 666	HAND2*	G/A	45.85	0.73	0.65 to 0.81	1.38×10^−8^	0.531
1	rs11579055	203 031 315	PPFIA4*	T/G	32.26	0.67	0.59 to 0.76	1.54×10^−10^	0.527
1	rs6694477	203 035 365	PPFIA4*	G/A	21.05	0.64	0.55 to 0.74	1.53×10^−9^	0.515
4	rs12507756	174 609 772	HAND2*	C/T	45.78	0.73	0.65 to 0.81	2.00×10^−8^	0.459
1	rs6689393	154 426 097	KCNN3*	A/G	45.99	1.07	0.96 to 1.19	0.199	0.458
1	rs535709	170 120 831	METTL11B*	C/T	12.92	1.04	0.88 to 1.22	0.648	0.430

*Genes are previously proven AF associated loci.

AF, atrial fibrillation; Chr., chromosome; GWAS, Genome-Wide Association Study; MAF, minor allele frequency; SNP, single-nucleotide polymorphism.

### CNN-GWAS performance after propensity-score matching

After 1:1 propensity-score matching, 862 AF patient group and 862 control group were compared with test the AF prediction power of CNN-GWAS (online supplemental table 5). After five repeated analyses with CNN-GWAS, the AUC values were 0.78±0.01 for Japanese, 0.78±0.01 for European at p<5.0×10^−8^, 0.78±0.01 for multiethnic group at p<1.0×10^−5^, respectively.

### Prediction results by PRS

We evaluated AF prediction power based on the PRS. The numbers of SNPs for PRS calculation by the p value cut-off are displayed in online supplemental table 6. The AUC values of Japanese and multiethnic groups were 0.82 and 0.83, respectively, at p<0.001. There were no significant differences compared with the predictive power of CNN-GWAS. However, the AUC value of European was 0.72 at p<1.0×10^−6^, which showed decreased predictive power compared with other models.

## Discussion

### Main findings

In this study, we explored whether a collaborative method of the CNN and GWAS was feasible in predicting the risk of AF based on the genetic data of a large population. The CNN-GWAS model achieved a reasonably acceptable AF prediction power (AUC 0.74~0.82) in the Korean population by utilising moderate AF-associated SNPs proven in three independent cohorts with different ethnicities. We verified the CNN-GWAS model by randomly shuffling the dataset five times, demonstrating no AF predictive power using SNPs with non-significant *P*-value subsets and no predictive powers for odd and even cohort registration numbers using genetic information. The predictive model of CNN-GWAS showed a stable predictive power compared with PRS even when GWAS summary statistics derived from other ancestry cohorts are applied to different ethnic cohorts. We also confirmed the high impact of pre-reported AF associated genetic loci on the AF prediction power in the CNN-GWAS model that were trained in the right direction by the Grad-CAM method. The CNN-GWAS algorithms capture the cumulative effects and genetic interactions of less significant or undiscovered SNPs that determine the manifestation of the AF phenotype.

### Emerging roles of the CNN in clinical cardiology

The use of AI, which enables a fast, sophisticated diagnosis, treatment and improved patient care workflow, and precision medical care, is increasing in clinical practice. AI is particularly useful for analysing data-rich technology-based objectives, such as omics, mobile device biometrics and electronic health records to obtain clinically useful information.^18^ The high predictive power of AI is also useful for cardiovascular disease, a slowly progressive disease with multifactorial pathophysiology and cardiac arrhythmia disease, which is difficult to predict, occurs suddenly and causes various complications.^19^ AI has been variously tested for the diagnostic purposes of cardiac diseases,^20^ and its high prognostic prediction power in cardiac imaging and electrocardiograms has already been verified.^21^ In this study, in combination with the GWAS data, AI demonstrated very high predictability of the common cardiac arrhythmia, AF, without including the clinical characteristics, personal habits or environmental factors. This further supports the evidence that AF is a heritable disease strongly affected by genetic factors.^4 5 22^

### Implications of CNN-GWAS-based precision medicine

In this study, we used AI algorithms of supervised learning techniques and the CNN, which is a deep learning method. Because a large well-curated clinical dataset is essential to properly train the deep learning,^23^ well quality-controlled genetic information has advantages over other clinical dataset. The advantages of deep learning are easy image recognition, no working memory limitations and its use with both supervised and non-supervised learning.^19^ On the other hand, the weaknesses of deep learning including the CNN are the possibility of overfitting and the error of learning when providing a biased training dataset. These two problems can be overcome by increasing the sample size of the training dataset or decreasing the number of hidden layers.^24^ The K-fold cross-validation is reported to be more accurate than the traditional split-sample approach.^25^ In this study, we used a single convolution and a fully connected layer and the K-fold cross-validation to evaluate over a half million genomic data of 6358 subjects. The output of the CNN-GWAS was verified in four different ways. Moreover, the black-box region, which is a chronic problem in the CNN analyses, was partially analysed by the Grad-CAM method, and AI calculations assigned high contribution scores to prediscovered AF associated genetic loci, especially *PITX2*. It is expected that the prudent monitoring of one AI algorithm by another AI algorithm will be used in the future.

### Study limitations

There are several potential limitations to our study. First, the results of this study cannot be generalised due to the nature of AI, which is greatly influenced by the training dataset. Second, this study included a highly selected group of patients (60 years old and younger) who were referred for AF ablation. This select patient population represents symptomatic antiarrhythmic drug-resistant early-onset AF. Third, the outcome of this study based on the Korean AF cohort data may not be generalised to other cohorts with different ethnicities and races. Fourth, the reason why a p≥0.99 was used for the SNP analysis of the non-significant p values used for validation of this study is due to the limitation of the computing power when it is executed at a p≥0.05. Fifth, the sample size of this study is relatively small compared with other large-scale GWAS studies. However, it satisfies the research purpose of evaluating the reproducibility of AF-associated SNPs after CNN application in the same patient group as our previous study proven by conventional statistical methods.

## Conclusions

In summary, the CNN-GWAS algorithm can be used to predict the AF, but comparison and verification with other models will be further warranted. The CNN-GWAS algorithms capture the cumulative effects and genetic interactions of moderately associated but statistically significant genes that determine the manifestation of the AF phenotype. AF can be predicted by genetic information alone with moderate accuracy.

## Data Availability

Data may be obtained from a third party and are not publicly available. Data contain sensitive patient information. Sharing of data is restricted by ethical approvals and the Personal Information Protection Act of the Republic of Korea. Access to data to reproduce results requires the application to and permission from Professor Hui-Nam Pak and The National Biobank of Korea.
