# Supplementary material for: Genome-wide association study-based prediction of atrial fibrillation using artificial intelligence
Source: Open Heart. 2022 Jan 27;9(1):e001898. doi: 10.1136/openhrt-2021-001898 (PMC8796259; doi:10.1136/openhrt-2021-001898)
Supplement: Supplementary data [file openhrt-2021-001898supp001.pdf]

Supplementary Materials

Genome-wide association study-based prediction of atrial fibrillation using artificial intelligence

Oh-Seok Kwon, PhD[a]; Myunghee Hong, PhD[a]; Tae-Hoon Kim, MD[a]; Inseok Hwang[a], Jaemin Shim, MD, PhD[b]; Eue-Keun Choi, MD, PhD[c]; Hong Euy Lim, MD, PhD[d]; Hee Tae Yu, MD, PhD[a]; Jae-Sun Uhm, MD, PhD[a]; Boyoung Joung, MD, PhD[a]; Seil Oh, MD, PhD[c]; Moon-Hyoung Lee, MD, PhD[a]; Young-Hoon Kim, MD, PhD[b]; Hui-Nam Pak, MD, PhD[a].

[a]*Yonsei University Health System, Seoul, Republic of Korea*

[b]*Korea University Cardiovascular Center, Seoul, Republic of Korea*

[c]*Seoul National University, Seoul, Republic of Korea*

[d]*Hallym University, Republic of Korea*

Kwon O-S, *et al. Open Heart* 2022; 9:e001898. doi: 10.1136/openhrt-2021-001898

Study design and subjects

We included 6,358 subjects from 4 independent cohorts and their GWAS data. The Korean AF Network is a consortium of four educational hospitals (Korea University Guro Hospital, Korea University Anam Hospital, Seoul National University Hospital, and Severance Hospital in the Yonsei University Health System) located in the Seoul area and concentrates on AF ablation related clinical studies. The Yonsei AF Ablation cohort and Korean AF Network were approved by the Institutional Review Board at each institution that participated in this study. Written informed consent was obtained from all patients.

As a control group, 5,486 samples were recruited from the health examinee (HEXA) cohort (n=3,700) and Korean Multi-Rural Communities Cohort Study (n=1,786, Figure 1). Both control cohorts are ongoing studies and a major part of the Korea Genome Epidemiology Study (KoGES) initiated in 2001. The HEXA cohort recruited from a large urban population and the Korean Multi-Rural Communities Cohort recruited volunteers older than 40 years of age living in a rural area. All subjects recruited were Korean, excluding all other ancestries. The informed consent requirement in the HEXA and Korean Multi-Rural Communities Cohort were waived.

Genotyping

The genetic datasets were genotyped with an Array 6.0 chip and genotype calls were identified by using the Birdseed V2 algorithm. The quality control (QC)[1, 2] was performed to the following criteria: (1) deviation from the Hardy-Weinberg equilibrium with a $P<1.0\times10^{-7}$, (2) minor allele frequency (MAF) < 1%, and (3) call rate < 95%. We additionally excluded ambiguous variants including the indel variants. An imputation analysis was not

performed in order to prevent overfitting and to reduce any uncertainty. The inflation factor λ was estimated to be 1.043 after merging all our cohorts (Supplementary Figure 1A). The final 531,766 SNPs were used for model training and the association analyses (Supplementary Figure 1B).

Network model design

CNN is an architecture for learning image data because that can filter spatial locality and capture the interactions between features using receptive fields.[3] Convolution operation, which plays an important role in CNN, performs feature extraction from images using optimizable parameters called kernel or filter. It has been widely applied in various fields, and it can adaptively learn spatial hierarchies of data features from low- to high-level patterns. The application of CNN was possible because SNP input data located in base pairs can be controlled with characteristics similar to images in that individual allele information is arranged on the same physical base pair.

The first convolutional layer used a 3×1 convolutional kernel because of the one-dimensional genetic information encoded by the linear DNA strands. The pooling layer was not added for testing each SNP sequentially with the null hypothesis of no association. The number of convolution filters used in the convolution layer was calculated according to the input size as in the following equation (1):

$$Conv\ \ K = max(n/8\,, 64), \qquad (1)$$

where n is the number of input SNPs and is designed to have a maximum of 64 kernels. As implied in the above equation, the first layer was led to the k-kernel to extract patterns of various features from the (X_1, X_2, ... X_n) input. In consideration of the protective effects with

dying neurons due to rare variants, the activation function was adopted as a leaky rectified linear unit (Leaky ReLU$(x) = max(0.1x, x)$). After the convolution layer, k-filtered patterns derived from the convolution layer were connected to the fully connected layer (FC) for the classification. Here, the FC activation function was applied with a rectified linear unit (ReLU)., and the number of neurons was obtained by the following equation (2):

$$FC\ \ neurons = max(n * K/8\,,1{,}024). \tag{2}$$

In the above equation, the FC layer is the determined maximum of 1,024 neurons by input SNPs and the number of filters. Finally, because the output layer was a probability for the phenotype, it was a probability from 0 to 1 by a sigmoid function. We implemented with Python (ver. 3.5) and TensorFlow (ver. 1.14.0) backend. Since the number of input SNPs differs depending on the *P*-value cutoff, the number of convolution filters and neurons of the FC layer were identified with a manual search. Using the mean square error and log-loss metrics, lightweight architecture was chosen unless a significant difference was found.

CNN-GWAS model training

The learning rate *Lr* began at 10^{-3}, where the loss decreased by 0.99 times below 2 and by 0.999 times below 1, eventually converging to 10^{-6} as the following equation (3):

$$Lr = \begin{cases} 10^{-3} & initial\ \ state \\ 0.99Lr & if\ \ loss < 2 \\ 0.999Lr & if\ \ loss < 1 \\ 10^{-6} & convergence\ \ state \end{cases} . \tag{3}$$

To avoid overfitting, the dropout rate was set to 10%, and early stopping was used to stop learning. If the loss was not continuously improved to less than 1% for the three epochs, learning was stopped. In addition, to add stability and improve the generalization, L1 and L2

regularization were both set to 10^{-5}. The hyperparameters (learning rate, dropout rate, L1, and L2 regularization) were selected with grid search.

The GWAS data showed that the control group was relatively larger than the case group. If this balance of the GWAS data is not considered, models are likely to be biased and trained into the control group. Therefore, we applied a stratified K-fold cross-validation technique that maintained a case/control ratio of 1:1 and induced the effect of cross-validation at the same time. The folding K used in the experiment was randomly determined from 5 to 10 for each epoch.

The loss function applied a sigmoid cross-entropy loss function for the binary classification. To train our network, to achieve minimum log-loss, the network weights were optimized using an Adam optimizer. The applied cross-validation and validation set was used to select appropriate hyper-parameters and find the optimal model for classification.

CNN-GWAS verification

To verify our model, four validation processes were conducted. First, we repeated the training, validation, and test processes 5-times to demonstrate the reproducibility of the AF prediction and each sample was randomly constructed. Second, to examine whether SNPs of statistically non-significant *P*-values by a logistic regression did not really affect the AF prediction, a SNP list was constructed and verified based on a $P \geq 0.99$. Third, in order to identify that there was no predictive power for a phenotype without heritability (here are odd-even registration numbers) other than AF, the validity was verified by replacing the AF label with an odd-even registration number. We chose the odd-even registration numbers to account for the unexpected bias by the random labels. Fourth, the saliency score of each SNP

for AF prediction was analyzed in all AF patients (n=872) using a model of best-performance among the model (Figure 2E). A Grad-CAM was applied to calculate the contribution score of each SNP for the AF prediction of the individual. To visualize the overall significance of SNPs contributing to the prediction, the saliency score analysis procedure was constructed with the following steps: (1) The SNP information of AF patients encoded by the additive model was sorted in a physical base-pair order, (2) AF patients that failed classification were excluded from feature visualization, (3) the saliency map was derived from 5 different models pre-trained with different sample configurations, taking into account the bias in the training phase, (4) the important SNPs contributing to AF prediction were calculated as the average of the saliency map for each patient, (5) the final mean saliency score was normalized, and (6) The threshold for the highlight of important features was set at 5%. The Grad-CAM equation (4) is described below:

$$Saliency\ \ score_i^c = \sum_k w_k^c A_i^c, \tag{4}$$

where the $Saliency\ \ score_i^c$ is the vector represented contribution of each SNP for the AF prediction of an individual, *i* is the location of the feature map A^k, *c* is the class, and w_k^c is the backpropagation gradient of the convolution kernel *k*. The mean contribution score of AF predictions was calculated as the mean of each saliency score of the individual SNPs in the predicted AF patients. Fifth, to identify whether the issue by class imbalance affected the AF prediction, we conducted a propensity-score matching study.

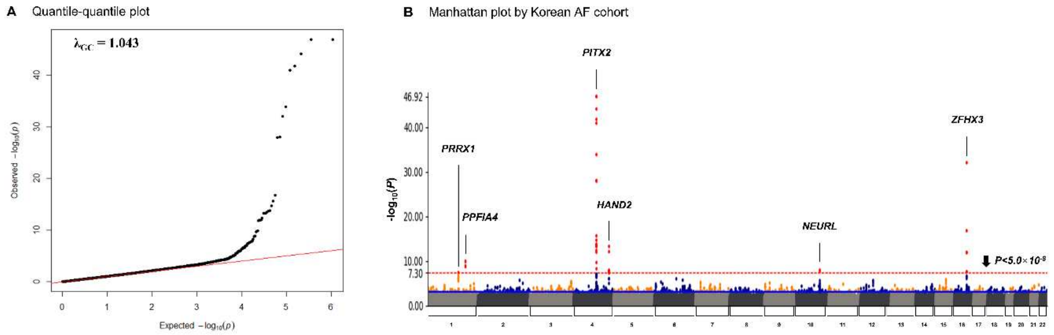

Supplementary Figure. 1. Quantile-quantile plot and Manhattan plot by GWAS of Korean AF cohort.

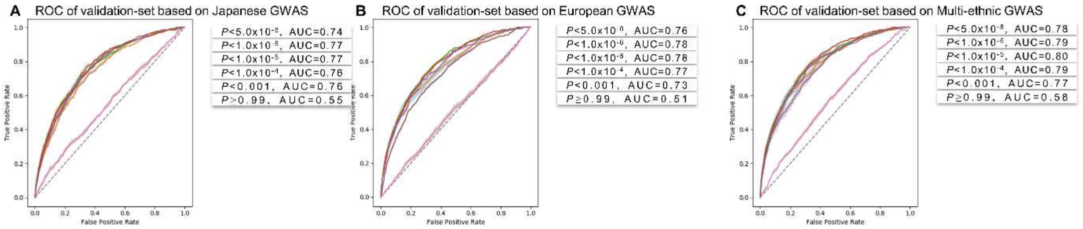

Supplementary Figure 2. Performance evaluation results for validation-set. (A-C) The results of the AF prediction at each *P*-value cutoff in each ethnic-specific validation set.

Supplementary Table 1. Baseline summary of previously reported ethnic-specific GWAS cohorts.

**Characteristics**	**Japanese** [4]	**European** [5]	**Multi-ethnic** [6]					
			**Total**	**EUR**	**ASN**	**AA**	**BRAZ**	**HISP**
**Cases, N**	8,180	60,620	65,446	55,114 (84.2%)	8,180 (12.5%)	1,307 (2.0%)	568 (0.9%)	277 (0.4%)
**Controls, N**	28,612	970,216	539,544	499,095 (92.5%)	28,612 (5.3%)	7,660 (1.4%)	1,096 (0.2%)	3,081 (0.6%)
**Overall, N**	36,792	1,030,836	604,990	554,209 (91.6%)	36,792 (6.1%)	8,967 (1.5%)	1,664 (0.3%)	3,358 (0.6%)

EUR, European; ASN, Asian; AA, African American; BRAZ, Brazilian; HISP, Hispanic.

Supplementary Table 2. The number of common SNPs by *P*-value cutoffs for each population type.

***P*-value cutoff**	**Population type**		
	**Japanese** [4]	**European** [5]	**Multi-ethinic** [6]
< 0.001	2,211	5,401	4,732
$< 1.0\times10^{-4}$	587	2,755	2,372
$< 1.0\times10^{-5}$	262	1,704	1,540
$< 1.0\times10^{-6}$	153	1,192	1,037
$< 5.0\times10^{-8}$	91	814	723
≥ 0.990	4,221	4,699	4,965

SNPs, single nucleotide polymorphisms.

Supplementary Table 3. Predictive performance for each best model by the SNP set derived from ethnic-specific GWAS.

**Population type**	***P*-value**	**SNPs**	**AUC**	**Sens**	**Spec**	**PPV**	**NPV**	**Gini**	**Log-loss**	**MSE**
Japanese	<0.001	2,211	0.793	0.789	0.688	0.287	0.953	0.585	0.735	0.133
European	$<1.0\times10^{-6}$	1,192	0.808	0.743	0.745	0.317	0.948	0.615	0.701	0.106
Multi-ethnic	$<1.0\times10^{-5}$	1,540	0.836	0.760	0.762	0.338	0.952	0.672	0.564	0.084

SNPs, single nucleotide polymorphisms; AUC, area under the curve; Sens, sensitivity; Spec, specificity; PPV, positive predictive value; NPV, negative predictive value; Gini, gini coefficient; MSE, mean square error.

BMJ Publishing Group Limited (BMJ) disclaims all liability and responsibility arising from any reliance placed on this supplemental material which has been supplied by the author(s)

Supplementary Table 4. Prediction performance of the AF associated SNP sets for the odd-even registration numbers.

Population type	*P*-value	SNPs	AUC	Sens	Spec	PPV	NPV	Gini	Log-loss	MSE
Japanese	<0.001	2,211	0.50±0.01	0.50±0.05	0.51±0.05	0.51±0.01	0.51±0.01	-0.01±0.02	0.87±0.01	0.31±0.02
	$<1.0\times10^{-4}$	587	0.51±0.01	0.53±0.04	0.51±0.02	0.52±0.01	0.52±0.02	0.03±0.03	0.75±0.01	0.26±0.01
	$<1.0\times10^{-5}$	262	0.50±0.02	0.53±0.03	0.49±0.03	0.51±0.01	0.51±0.01	0.01±0.03	0.73±0.01	0.25±0.01
	$<1.0\times10^{-6}$	153	0.51±0.01	0.55±0.02	0.49±0.03	0.52±0.01	0.52±0.01	0.02±0.02	0.71±0.01	0.26±0.01
	$<5.0\times10^{-8}$	91	0.49±0.00	0.53±0.06	0.47±0.06	0.50±0.00	0.50±0.01	-0.01±0.01	0.70±0.00	0.25±0.00
European	<0.001	5,401	0.50±0.01	0.49±0.04	0.52±0.04	0.51±0.01	0.50±0.01	0.00±0.02	0.99±0.05	0.27±0.04
	$<1.0\times10^{-4}$	2,755	0.48±0.01	0.48±0.02	0.50±0.02	0.49±0.01	0.49±0.01	-0.04±0.01	0.87±0.01	0.28±0.03
	$<1.0\times10^{-5}$	1,704	0.48±0.01	0.49±0.07	0.50±0.08	0.50±0.01	0.49±0.01	-0.04±0.03	0.84±0.01	0.28±0.02
	$<1.0\times10^{-6}$	1,192	0.49±0.01	0.48±0.05	0.52±0.06	0.51±0.01	0.50±0.01	-0.02±0.02	0.79±0.01	0.26±0.02
	$<5.0\times10^{-8}$	814	0.50±0.01	0.53±0.05	0.47±0.03	0.50±0.01	0.50±0.01	-0.01±0.02	0.76±0.01	0.26±0.01
Multi-ethnic	<0.001	4,732	0.49±0.01	0.52±0.06	0.49±0.07	0.5±0.02	0.50±0.02	-0.02±0.03	2.61±1.05	0.46±0.26
	$<1.0\times10^{-4}$	2,372	0.48±0.02	0.50±0.02	0.47±0.01	0.49±0.01	0.49±0.01	-0.04±0.03	0.83±0.03	0.26±0.01
	$<1.0\times10^{-5}$	1,540	0.49±0.01	0.45±0.01	0.56±0.01	0.51±0.00	0.50±0.00	-0.02±0.02	0.84±0.05	0.28±0.01
	$<1.0\times10^{-6}$	1,037	0.50±0.01	0.55±0.03	0.47±0.02	0.51±0.01	0.51±0.01	0.01±0.03	0.82±0.02	0.27±0.01
	$<5.0\times10^{-8}$	723	0.49±0.01	0.49±0.04	0.51±0.05	0.50±0.01	0.50±0.01	-0.02±0.02	0.87±0.02	0.28±0.00

Data are shown as the mean ± SD.

SNP, single nucleotide polymorphism; AUC, area under the curve; Sens, sensitivity; Spec, specificity; PPV, positive predictive value; NPV, negative predictive value; Gini, gini coefficient; MSE, mean square error.

Supplementary Table 5. Characteristics of case and control groups after 1:1 propensity-score matching.

Baseline characteristics	Case (N = 862)	Control (N = 862)	*P*-value
Age, year	50.4 ± 7.9	51.1 ± 8.0	0.070
Male sex, %	695 (80.6)	694 (80.5)	0.951
Body mass index, kg/m^2	25.0 ± 3.0	25.1 ± 3.0	0.908
Hypertension, %	299 (34.7)	268 (31.1)	0.112
Diabetes, %	65 (7.5)	69 (8.0)	0.719
Coronary artery disease, %	76 (8.8)	64 (7.4)	0.290
Stroke, %	54 (6.2)	49 (5.7)	0.684

Data are shown as the mean ± SD or n (%);

Supplementary Table 6. The number of SNPs for PRS calculation in each GWAS.

P-value cutoff*	# of non-missing alleles used for scoring		
	Japanese	European	Multi-ethnic
$< 5.0\times10^{-8}$	30	330	296
$< 1.0\times10^{-6}$	62	482	414
$< 1.0\times10^{-5}$	112	714	604
$< 1.0\times10^{-4}$	318	1,218	1,020
< 0.001	1,512	2,848	2,458
≥ 0.99	376	504	480

* r^2 was set to 0.1 in all ethnicities.

Supplementary References

1 Anderson CA, Pettersson FH, Clarke GM, et al. Data quality control in genetic case-control association studies. *Nat Protoc* 2010;**5**:1564-73.
2 Winkler TW, Day FR, Croteau-Chonka DC, et al. Quality control and conduct of genome-wide association meta-analyses. *Nat Protoc* 2014;**9**:1192-212.
3 Low SK, Takahashi A, Ebana Y, et al. Identification of six new genetic loci associated with atrial fibrillation in the Japanese population. *Nat Genet* 2017;**49**:953-8.
4 Nielsen JB, Thorolfsdottir RB, Fritsche LG, et al. Biobank-driven genomic discovery yields new insight into atrial fibrillation biology. *Nat Genet* 2018;**50**:1234-9.
5 Roselli C, Chaffin MD, Weng LC, et al. Multi-ethnic genome-wide association study for atrial fibrillation. *Nat Genet* 2018;**50**:1225-33.
